# Association Between Longitudinal Change in Abnormal Fasting Blood Glucose Levels and Outcome of COVID-19 Patients Without Previous Diagnosis of Diabetes

**DOI:** 10.3389/fendo.2021.640529

**Published:** 2021-03-30

**Authors:** Siwei Song, Shujing Zhang, Zhihui Wang, Sufei Wang, Yanling Ma, Pei Ma, Huilin Luo, Mengyuan Wang, Yang Jin

**Affiliations:** ^1^ Department of Respiratory and Critical Care Medicine, NHC Key Laboratory of Pulmonary Diseases, Union Hospital, Tongji Medical College, Huazhong University of Science and Technology, Wuhan, China; ^2^ Department of Scientific Research, Union Hospital, Tongji Medical College, Huazhong University of Science and Technology, Wuhan, China; ^3^ Department of Anesthesia, Wuhan Red Cross Hospital, Wuhan, China; ^4^ Department of Endocrinology, Union Hospital, Tongji Medical College, Huazhong University of Science and Technology, Wuhan, China

**Keywords:** fasting blood glucose trajectory, COVID-19, glycemic control, SARS-CoV-2, longitudinal change

## Abstract

This retrospective study examined changes in fasting blood glucose (FBG) levels during hospitalization and their effect on risk of death for Coronavirus disease 2019 (COVID-19) patients without previously diagnosed diabetes. A model with low- and high-stable pattern trajectories was established based on a longitudinal change in FBG levels. We analyzed FBG trajectory-associated clinical features and risk factors for death due to COVID-19. Of the 230 enrolled patients, 44 died and 87.83% had a low-stable pattern (average FBG range: 6.63–7.54 mmol/L), and 12.17% had a high-stable pattern (average FBG range: 12.59–14.02 mmol/L). There were statistical differences in laboratory findings and case fatality between the two FBG patterns. Multivariable logistic regression analysis showed that increased neutrophil count (odds ratio [OR], 25.43; 95% confidence interval [CI]: 2.07, 313.03), elevated direct bilirubin (OR, 5.80; 95%CI: 1.72, 19.58), elevated creatinine (OR, 26.69; 95% CI: 5.82, 122.29), lymphopenia (OR, 8.07; 95% CI: 2.70, 24.14), and high-stable FBG pattern (OR, 8.79; 95% CI: 2.39, 32.29) were independent risk factors for higher case fatality in patients with COVID-19 and hyperglycemia but no history of diabetes. FBG trajectories were significantly associated with death risk in patients with COVID-19 and no diabetes.

## Introduction

Coronavirus disease 2019 (COVID-19), caused by severe acute respiratory syndrome coronavirus 2, has become a major threat to global health since it was first reported in December 2019. As of February 16, 2021, more than 100 million cases of COVID-19 have been confirmed worldwide, resulting in more than 2.4 million deaths (1). Pneumonia is the main clinical manifestation of COVID-19. Mild cases may have no obvious symptoms, whereas severe cases may involve severe respiratory distress syndrome, septic shock, or multiple organ dysfunction syndrome (2).

It is well-known that hyperglycemia and poor glycemic control are established risk factors for most infectious diseases (3, 4), including severe acute respiratory syndrome (5) and Middle East respiratory syndrome (6). Hyperglycemia is detrimental to the control of viremia and inflammation and aggravates morbidity and mortality in patients. Previous studies have shown that stress hyperglycemia due to an acute blood glucose disorder can occur in patients with COVID-19, even in those without a previous diagnosis of diabetes. Furthermore, it has been confirmed that hyperglycemia is an independent poor prognostic factor for COVID-19 with or without pre-existing diabetes (7, 8).

Long-term hyperglycemia may induce abnormal coagulation function, endothelial dysfunction, and inflammatory cytokine overproduction caused by abnormal immune activation (9, 10). Therefore, blood glucose control is necessary for recovery from infectious diseases. Evidence indicates that poor glycemic control in patients with COVID-19 is associated with a higher risk of complications or case fatality (11–13). However, these studies, in which fasting blood glucose (FBG) was presented in the form of an initial value at admission or as a mean value during hospitalization, have primarily focused on patients with pre-existing diabetes. Moreover, they did not consider longitudinal change in FBG related to COVID-19. Therefore, in this study, we aimed to examine the association between longitudinal change in COVID-19-related abnormal FBG and outcome for patients with COVID-19 who without a previous diagnosis of diabetes.

## Material and Methods

### Study Design and Participants

This retrospective study was conducted in three hospitals in Wuhan, China, namely, Wuhan Union Hospital, Wuhan Union West Hospital, and Wuhan Red Cross Hospital, all of which had been mandatorily designated to treat patients with COVID-19. Ethical approval was granted by the Institutional Ethics Committees of Wuhan Union Hospital (No. 0036). No patients or medical staff participated in the study design or statistical analysis.

**Figure 1:**
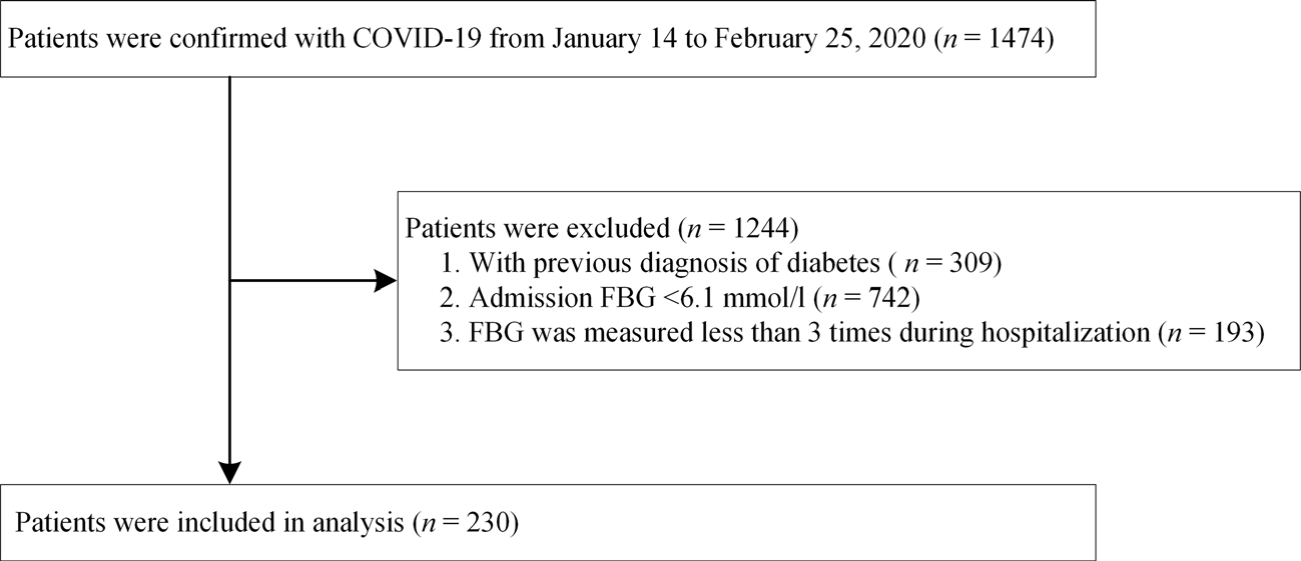
Flowchart of patient selection.

A total of 1,474 adult patients with a laboratory-confirmed diagnosis of COVID-19 had been admitted to one of the three hospitals between January 14 and February 25, 2020. A diagnosis of COVID-19 was laboratory-confirmed in accordance with the interim guidance formulated by the World Health Organization (14). We excluded 1,244 patients based on the following exclusion criteria: (i) previous diagnosis of diabetes; (ii) admission FBG <6.1 mmol/L (impaired fasting glucose was defined as FBG >6.1 mmol/L according to the World Health Organization guidelines (15); (iii) FBG measured less than three times during hospitalization. Finally, 230 patients with COVID-19 were included in the analysis. A patient selection flowchart is presented in **Figure 1**. All patients were followed up until discharge or in-hospital death.

### Data Collection

Information derived from demographic data, clinical manifestations, comorbidities, laboratory findings during hospitalization, and final outcomes (discharge or death) of all enrolled patients was acquired retrospectively from the admission records of the relevant departments. Comorbidities included chronic obstructive pulmonary disease, asthma, hypertension, chronic cardiac disease, anemia, chronic kidney disease, chronic hepatic disease, cerebrovascular disease, and malignant disease, all of which had been diagnosed using standard criteria. Laboratory findings included complete blood count, coagulation profile, renal and liver function, and inflammation markers.

Clinical management was standardized and appeared to be similar across the three hospitals, comprising antiviral therapy; symptomatic and supportive treatment; empirical antimicrobial therapy as appropriate to prevent or treat secondary infections; and oxygen support, in accordance with the Diagnosis and Treatment Plan for COVID-19 issued by the National Health Commission of People’s Republic of China (6^th^ Edition) (16). Hypoglycemic therapy included oral hypoglycemic medications and insulin injections. Patients were discharged at the discretion of the attending physician after a standard prescribed set of discharge criteria had been met, according to the aforementioned Diagnosis and Treatment Plan (16).

### Measurement of FBG

The timing and frequency of FBG monitoring varied from person to person, depending on hyperglycemia severity, and patients with severe hyperglycemia had more frequent FBG surveillance accordingly. In our study, we collected FBG data on at least three occasions during hospitalization, including at admission and at discharge. All blood samplings had been conducted under fasting conditions (overnight fasting for at least 8 hours). Serum concentrations of FBG had been measured using an automatic biochemical analyzer (AU5800 Analyzer; Beckman Coulter, Brea, CA).

### Statistical Analysis

**Figure 2:**
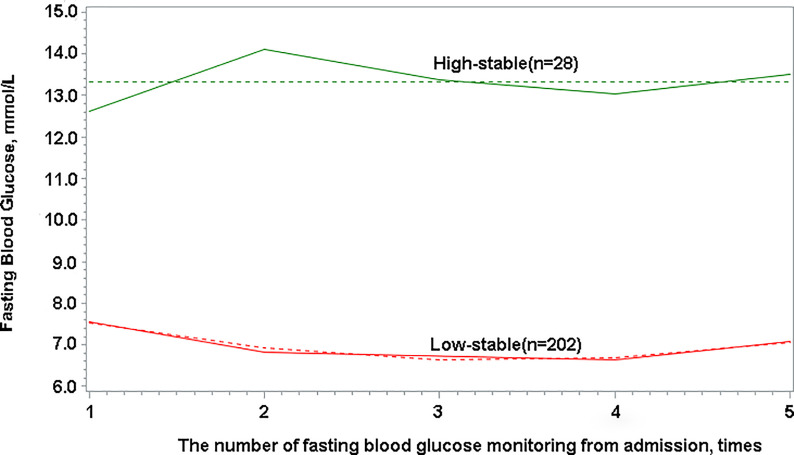
Fasting blood glucose trajectories with low-stable and high-stable patterns based on longitudinal change in fasting blood glucose.

FBG trajectories were modeled using group-based trajectory modeling (GBTM) for all enrolled participants with the FBG measured more than three times, using SAS Proc Traj program. GBTM, a form of finite mixed model, is applied to longitudinal data to identify sharing common potential development trajectories of group using maximum likelihood estimation, which has been increasingly applied in clinical research (17, 18). One advantage of GBTM is that its trajectories are not restricted to a single pattern since GBTM does not assume a single functional form of population, that is, it allows for group trajectories to rise, fall, or remain stable. Optimal trajectory numbers in GBTM cannot be selected and identified in advance; however, they can be identified according to superior and inferior models in the process of statistical analysis (17, 19). After exploring various numbers of potential trajectories and the polynomial orders of each trajectory, the optimal trajectory model was selected, which had the smallest Bayesian information criterion value. Moreover, the number of observed objects in each trajectory was guaranteed to account for at least 5% of the total number of cases, and the average posterior probability of each trajectory was >70% (18). Finally, two FBG trajectories were obtained in this study (**Figure 2**). The optimal FBG trajectory model was a polynomial order 2,0 model. Patients were categorized as having either “low-stable pattern” or “high-stable pattern” and two patient groups were formed accordingly.

Descriptive statistics were used to describe patient baseline data. Categorical variables are presented as numbers with percentage proportions, and continuous variables are expressed as mean ± standard deviation, if they were normally distributed, or as median (interquartile range [IQR]), if they were non-normally distributed. Proportions for categorical variables were compared using the *χ2* test, Cochran-Mantel-Hensel *χ2* test, or Fisher’s exact test. All laboratory data are presented as average concentrations based on measurements during hospitalization, including at admission and at discharge. The optimal cut-off value was determined using Youden’s index. In the descriptive analysis, the characteristics of the data were compared according to survival status and trajectory classes.

We conducted univariable and multivariable logistics regression analysis to identify factors correlating with case fatality, whose variables were screened using least absolute shrinkage and selection operator (LASSO) regression analysis to avoid overfitting for the large number of variables. A Kaplan-Meier survival curve was plotted to determine the relationship between the FBG patterns and the final outcomes. Linear mixed model was used to describe the relationship between changes in FBG levels and COVID-19 survivors and non-survivors. We analyzed the association between reduction in FBG level during hospitalization and prognosis using logistical regression. Pearson correlation coefficient was used to evaluate the correlation between glycemic variations and the evolution of biochemical parameters.

R software (version 3.6.3) was used to calculate Pearson’s correlation coefficient, and other analyses were conducted using SAS version 9.4 software.

## Results

### Demographics and Clinical Features of Patients

From January 14 to February 25, 2020, 1,474 patients with confirmed COVID-19 had been admitted to the aforementioned three hospitals. After excluding 309 patients with a previous diagnosis of diabetes, 742 with an FBG <6.1 mmol/L at admission, and 193 whose FBG had been measured less than three times during hospitalization, a total of 230 patients were retrospectively enrolled in final analysis (**Figure 1**). All of these patients had hyperglycemia during hospitalization (average FBG, 7.70 mmol/L).

The baseline data and clinical features of all patients are shown in **Table 1**. The median age of these patients was 63 (IQR, 54–70) years, and 139 (59.13%) were men. Among the 230 patients, 44 (19.13%) died during hospitalization. Compared with survivors, there were more elderly patients (63.64% vs. 35.48%, *P* = 0.0006) among non-survivors. Moreover, non-survivors were more likely to present expectoration (55.00% vs. 37.58%, *P* = 0.0444), have a history of malignant disease (15.00% vs. 4.46%, *P* = 0.0165), and require invasive mechanical ventilation (IMV) (38.64% vs. 4.84%, *P* < 0.0001).

**Table 1:** Baseline clinical characteristics and laboratory findings concerning patients with COVID-19 and hyperglycemia without previous diagnosis of diabetes.

Variables	All patients (n = 230)	Non-survivor (n = 44)	Survivor (n = 186)	*P*-value
Age, years				
Median, (IQR)	63.00 (54.00, 70.00)	69.00 (61.00, 73.50)	62.00 (52.00, 69.00)	0.0016
≤65, n (%)	136 (59.13)	16 (36.36)	120 (64.52)	0.0006
>65, n (%)	94 (40.87)	28 (63.64)	66 (35.48)	
Sex				
Female, n (%)	91 (39.57)	14 (31.82)	77 (41.40)	0.2426
Male, n (%)	139 (60.43)	30 (68.18)	109 (58.60)	
Onset symptoms				
Fever, n (%)	183/218 (83.94)	31/41 (75.61)	152/177 (85.88)	0.1067
Pharyngalgia, n (%)	8/201 (3.98)	2/38 (5.26)	6/163 (3.68)	0.6475
Fatigue, n (%)	100/206 (48.54)	21/39 (53.85)	79/167 (47.31)	0.4618
Muscular soreness, n (%)	42/203 (20.69)	12/38 (31.58)	30/165 (18.18)	0.0661
Cough, n (%)	158/210 (75.24)	27/40 (67.50)	131/170 (77.06)	0.2076
Expectoration, n (%)	84/205 (40.98)	22/40 (55.00)	62/165 (37.58)	0.0444
Hemoptysis, n (%)	6/202 (2.97)	0	6/164 (3.66)	0.5965
Dyspnea, n (%)	81/204 (39.71)	19/40 (47.50)	62/164 (37.80)	0.2612
Rhinorrhea, n (%)	3/202 (1.49)	1/40 (2.50)	2/162 (1.23)	0.4861
Nausea, n (%)	17/203 (8.37)	3/38 (7.89)	14/165 (8.48)	> 0.9999
Vomiting, n (%)	14/203 (6.90)	2/38 (5.26)	12/165 (7.27)	>0.9999
Stomach ache, n (%)	4/203 (1.97)	1/39 (2.56)	3/164 (1.83)	0.5770
Diarrhea, n (%)	29/204 (14.22)	6/40 (15.00)	23/164 (14.02)	0.8741
Poor appetite, n (%)	36/203 (17.73)	4/39 (10.26)	32/164 (19.51)	0.1738
Headache, n (%)	11/202 (5.45)	1/40 (2.50)	10/162 (6.17)	0.6964
Delirium, n (%)	0	0	0	0.3593
Chest distress, n (%)	66/204 (32.35)	17/40 (42.50)	49/164 (29.88)	0.1260
Non-symptoms, n (%)	0	0	0	0.1260
Comorbidity				
Chronic obstructive pulmonary disease, n (%)	3/198 (1.52)	1/40 (2.50)	2/158 (1.27)	0.4938
Asthma, n (%)	3/198 (1.52)	0	3/158 (1.90)	>0.9999
Hypertension, n (%)	74/202 (36.63)	15/40 (37.50)	59/162 (36.42)	0.8989
Chronic cardiac disease, n (%)	27/201 (13.43)	4/40 (10.00)	23/161 (14.29)	0.4768
Anemia, n (%)	1/197 (0.51)	1/40 (2.50)	0	0.2030
Chronic kidney disease, n (%)	9/197 (4.57)	3/40 (7.50)	6/157 (3.82)	0.3908
Chronic hepatic disease, n (%)	5/197 (2.54)	0	5/157 (3.18)	0.5852
Cerebrovascular disease, n (%)	6/197 (3.05)	2/40 (5.00)	4/157 (2.55)	0.6034
Malignant disease, n (%)	13/197 (6.60)	6/40 (15.00)	7/157 (4.46)	0.0165
Respiratory support				
Invasive mechanical ventilation, n (%)	26/230 (11.30)	17/44 (38.64)	9/186 (4.84)	<0.0001
Treatment				
Traditional Chinese medicine, n (%)	132/230 (57.39)	14/44 (31.81)	118/230 (63.44)	0.0001
Antiviral therapy, n (%)	210/230 (91.30)	40/44 (90.91)	170/186 (91.40)	<0.9999
Antibiotic therapy, n (%)	175/230 (76.09)	40/44 (90.91)	135/186 (72.58)	0.0180
Corticosteroid, n (%)	93/230 (40.43)	26/44 (59.09)	67/186 (36.02)	0.0050
Intravenous immunoglobin, n (%)	58/230 (25.22)	12/44 (27.27)	46/186 (24.73)	0.7270
Antihypertensive medicine, n (%)	46/230 (20.00)	3/44 (6.82)	43/186 (23.12)	0.0263
Lipid-lowering therapy, n (%)	11/230 (4.78)	1/44 (2.27)	10/186 (5.38)	0.6350
Hypoglycemic therapy, n (%)	11/230 (4.78)	6/44 (13.64)	5/186 (2.69)	0.0022
Laboratory findings				
Fasting blood glucose, mmol/L				
Median, (IQR)	6.79 (6.05, 8.49)	9.35 (8.13, 11.97)	6.49 (5.92, 7.52)	<0.0001
Low-stable, n (%)	202/230 (87.83)	27/44 (61.36)	175/186 (94.09)	<0.0001
High-stable, n (%)	28/230 (12.17)	17/44 (38.64)	11/186 (5.91)	
White blood cell count, ×10^9^ /L				0.0002
≤5.6, n (%)	63/230 (27.39)	2/44 (4.55)	61/186 (32.80)	
>5.6, n (%)	167/230 (72.61)	42/44 (95.45)	125/186 (67.20)	
Red blood cell count, ×10^12^ /L				0.1443
≤4.0, n (%)	129/230 (56.09)	29/44 (65.91)	100/186 (53.76)	
>4.0, n (%)	101/230 (43.91)	15/44 (34.09)	86/186 (46.24)	
Platelet count, ×10^9^ /L				<0.0001
≤136, n (%)	45/230 (19.57)	23/44 (52.27)	22/186 (11.83)	
>136, n (%)	185/230 (80.43)	21/44 (47.73)	164/186 (88.17)	
Neutrophil count, ×10^9^ /L				<0.0001
≤4.7, n (%)	92/230 (40.00)	2/44 (4.55)	90/186 (48.39)	
>4.7, n (%)	138/230 (60.00)	42/44 (95.45)	96/186 (51.61)	
Lymphocyte count, ×10^9^ /L				<0.0001
≤0.7, n (%)	62/230 (26.96)	33/44 (75.00)	29/186 (15.59)	
>0.7, n (%)	168/230 (73.04)	11/44 (25.00)	157/186 (84.41)	
Monocyte count, ×10^9^ /L				<0.0001
≤0.2, n (%)	14/230 (6.09)	10/44 (22.73)	4/186 (2.15)	
>0.2, n (%)	216/230 (93.91)	34/44 (77.27)	182/186 (97.85)	
Eosinophil count, ×10^9^ /L				<0.0001
≤0.01, n (%)	23/230 (10.00)	13/44 (29.55)	10/186 (5.38)	
>0.01, n (%)	207/230 (90.00)	31/44 (70.45)	176/186 (94.62)	
Basophil count, ×10^9^ /L				<0.0001
≤0.01, n (%)	38/230 (16.52)	7/44 (15.91)	31/186 (16.67)	
>0.01, n (%)	192/230 (83.48)	37/44 (84.09)	155/186 (83.33)	
Total bilirubin, µmol/L				<0.0001
≤12.3, n (%)	113/230 (49.13)	9/44 (20.45)	104/186 (55.91)	
>12.3, n (%)	117/230 (50.87)	35/44 (79.55)	82/186 (44.09)	
Direct bilirubin, µmol/L				<0.0001
≤4.6, n (%)	137/230 (59.57)	7/44 (15.91)	130/186 (69.89)	
>4.6, n (%)	93/230 (40.43)	37/44 (84.09)	56/186 (30.11)	
Alanine aminotransferase, U/L				0.4284
≤23.3, n (%)	25/230 (10.87)	3/44 (6.82)	22/186 (11.83)	
>23.3, n (%)	205/230 (89.13)	41/44 (93.18)	164/186 (88.17)	
Aspartate aminotransferase, U/L				0.0313
≤27.2, n (%)	61/230 (26.52)	6/44 (13.64)	55/186 (29.57)	
>27.2, n (%)	169/230 (73.48)	38/44 (86.36)	131/186 (70.43)	
Alkaline phosphatase, U/L				0.0014
≤53, n (%)	44/228 (19.30)	1/44 (2.27)	43/184 (23.37)	
>53, n (%)	184/228 (80.70)	43/44 (97.73)	141/184 (76.63)	
Glutamyl transpeptidase, U/L				0.9278
≤27.3, n (%)	53/228 (23.25)	10/44 (22.73)	43/184 (23.37)	
>27.3, n (%)	175/228 (76.75)	34/44 (77.27)	141/184 (76.63)	
Total protein, g/L				<0.0001
≤58.6, n (%)	59/230 (25.65)	23/44 (52.27)	36/186 (19.35)	
>58.6, n (%)	171/230 (74.35)	21/44 (47.73)	150/186 (80.65)	
Globulin, g/L				0.4592
≤30.5, n (%)	95/230 (41.30)	16/44 (36.36)	79/186 (42.47)	
>30.5, n (%)	135/230 (58.70)	28/44 (63.64)	107/186 (57.53)	
Prealbumin, mg/L				<0.0001
≤100.6, n (%)	37/210 (17.62)	24/44 (54.55)	13/166 (7.83)	
>100.6, n (%)	173/210 (82.38)	20/44 (45.45)	153/166 (92.17)	
Albumin, g/L				<0.0001
≤33.8, n (%)	163/230 (70.87)	44/44 (100.00)	119/186 (63.98)	
>33.8, n (%)	67/230 (29.13)	0	67/186 (36.02)	
Total bile acid, µmol/L				0.0695
≤3.5, n (%)	95/228 (41.67)	13/44 (29.55)	82/184 (44.57)	
>3.5, n (%)	133/228 (58.33)	31/44 (70.45)	102/184 (55.43)	
Creatinine, µmol/L				<0.0001
≤111, n (%)	203/230 (88.26)	24/44 (54.55)	179/186 (96.24)	
>111, n (%)	27/230 (11.74)	20/44 (45.45)	7/186 (3.76)	
Blood Urea Nitrogen, mmol/L				<0.0001
≤8.2, n (%)	174/230 (75.65)	14/44 (31.82)	160/186 (86.02)	
>8.2, n (%)	56/230 (24.35)	30/44 (68.18)	26/186 (13.98)	
Uric acid, µmol/L				0.0001
≤428, n (%)	217/230 (94.35)	36/44 (81.82)	181/186 (97.31)	
>428, n (%)	13/230 (5.65)	8/44 (18.18)	5/186 (2.69)	
Creatine kinase, U/L				0.0002
≤83, n (%)	108/214 (50.47)	11/44 (25.00)	97/170 (57.06)	
>83, n (%)	106/214 (49.53)	33/44 (75.00)	73/170 (42.94)	
D-dimer, µg/mL				<0.0001
≤0.97, n (%)	84/223 (37.67)	2/43 (4.65)	82/180 (45.56)	
>0.97, n (%)	139/223 (62.33)	41/43 (95.35)	98/180 (54.44)	
Prothrombin time, s				<0.0001
≤14.3, n (%)	158/223 (70.85)	13/43 (30.23)	145/180 (80.56)	
>14.3, n (%)	65/223 (29.15)	30/43 (69.77)	35/180 (19.44)	
International Normalized Ratio				<0.0001
≤1.1, n (%)	141/223 (63.23)	13/43 (30.23)	128/180 (71.11)	
>1.1, n (%)	82/223 (36.77)	30/43 (69.77)	52/180 (28.89)	
Activated partial thromboplastin time, s				<0.0001
≤40.2, n (%)	164/226 (72.57)	19/43 (44.19)	145/183 (79.23)	
>40.2, n (%)	62/226 (27.43)	24/43 (55.81)	38/183 (20.77)	
Thromboplastin time, s				0.0123
≤16.5, n (%)	164/218 (75.23)	26/43 (60.47)	138/175 (78.86)	
>16.5, n (%)	54/218 (24.77)	17/43 (39.53)	37/175 (21.14)	
Fibrinogen, g/L				0.0568
≤4.1, n (%)	124/226 (54.87)	18/43 (41.86)	106/183 (57.92)	
>4.1, n (%)	102/226 (45.13)	25/43 (58.14)	77/183 (42.08)	
C-reactive protein, mg/L				<0.0001
≤21.4, n (%)	83/224 (37.05)	0	83/180 (46.11)	
>21.4, n (%)	141/224 (62.95)	44/44 (100.00)	97/180 (53.89)	
Erythrocyte sedimentation rate, mm/h				0.6486
≤22, n (%)	10/91 (10.99)	2/14 (14.29)	8/77 (10.39)	
>22, n (%)	81/91 (89.01)	12/14 (85.71)	69/77 (89.61)	

Data are median (IQR) or n (%). P-values were calculated using χ^2^ test, Cochran–Mantel–Haenszel χ^2^ test, Fisher’s exact test or Wilcoxon rank-sum test, as appropriate.

IQR, interquartile range.

In terms of treatment, the use of antibiotic (90.91% vs. 72.58%, *P* = 0.018), corticosteroid (59.09% vs. 36.02%, *P* = 0.0050), and hypoglycemic therapy (13.64% vs. 2.69%, *P* = 0.0022) was more common among non-survivors than survivors, whereas the use of traditional Chinese medicine (63.44% vs. 31.81, *P* = 0.0001) and antihypertensive medicine (23.12% vs. 6.82%, *P* = 0.0263) was more common in survivors than in non-survivors. There were no significant differences in the use of antiviral (*P* > 0.9999) and lipid-lowing therapy (*P* = 0.6350) between survivors and non-survivors.

The results of relevant laboratory examinations are shown in **Table 1**. We found that, compared with survivors, non-survivors were more likely to have abnormal routine blood test results, elevated hepatic and renal biochemical indicators, hypoproteinemia, and coagulation function disorders. The FBG in non-survivors (median, 9.35; IQR, 8.13–11.97) were higher than those in survivors (median, 6.49; IQR, 5.92–7.52; *P* < 0.0001).

### FBG Trajectories of Patients

The study population was categorized based on two observed trajectories of FBG during hospitalization. These trajectories were based on FBG levels and changing patterns (**Figure 2**): 87.83% (n = 202) of patients with sustained abnormal but relatively low FBG levels (categorized as “low-stable pattern”, in which the mean FBG levels ranged from 6.63 to 7.54 mmol/L during hospitalization), and 12.17% (n = 28) of patients with sustained high FBG levels (categorized as “high-stable pattern”, in which the mean FBG levels ranged from 12.59 to 14.02 mmol/L during hospitalization). The proportion of non-surviving patients with a high-stable pattern was 38.64%, which was found to be considerably higher than that of survivors (5.91%; *P* < 0.0001) (**Table 1**).

### Clinical Features Between Patients With Low- or High-Stable FBG Patterns

The clinical features concerning all patients with low- or high-stable FBG patterns are presented in **Supplementary Table 1**. Compared with patients having the low-stable pattern, those with the high-stable pattern were found to be older (median age, 69.50 vs 63.00 years; *P* = 0.0359) and to have a malignant disease (17.39% vs. 5.17%, *P* = 0.0265). There were no statistically significant differences in terms of onset symptoms and comorbidities, except for the presence of malignant disease, between the low- and the high-stable pattern groups. However, individuals with the high-stable pattern were more likely to accept IMV treatment throughout their whole course than those with the low-stable pattern (28.57% vs. 8.91%, *P* = 0.0021). Furthermore, in patients with the high-stable pattern, the use of corticosteroid (64.29% vs. 37.13%, *P* = 0.0061) and hypoglycemic therapy (39.29% vs. 0, *P* < 0.0001) was more common than in those with low-stable pattern.

**Figure 3:**
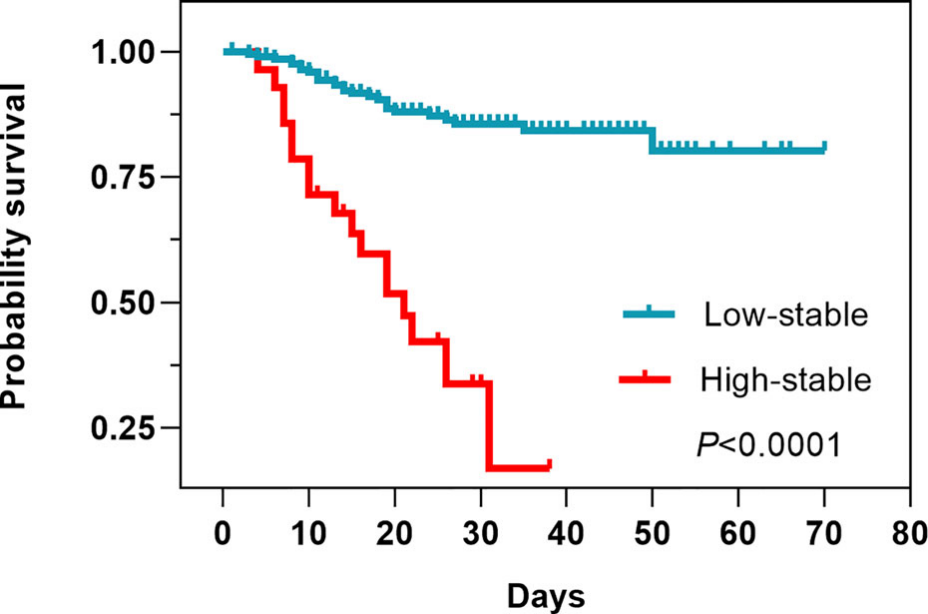
Survival analysis between low-stable and high-stable pattern trajectories.

Laboratory findings indicated several differences between the two FBG pattern groups. The high-stable pattern group had higher blood cell and neutrophil counts than the low-stable pattern group (96.43% vs. 69.31%*, P* = 0.0026; 96.43% vs. 54.95%, *P* < 0.0001, respectively), but lower platelet, lymphocyte, and monocyte counts (53.57% vs. 14.85%, *P* < 0.0001; 57.14% vs. 22.77%, *P* = 0.0001; 21.43% vs. 3.96%, *P* = 0.0001, respectively). Compared with patients in the low-stable pattern group, those in the high-stable pattern had a higher level of direct bilirubin (DBIL) (64.29% vs. 37.13%, *P* = 0.0061), a higher prothrombin time (57.14% vs. 33.85%, *P* = 0.0005), and a lower level of total protein (60.71% vs. 20.79%, *P <* 0.0001*).* In addition, 92.59% of patients in the high-stable pattern group had a high C-reactive protein level, which was significantly higher than in patients in the low-stable pattern group (58.88%, *P* = 0.0007). The survival analysis showed that the high-stable pattern was significantly associated with increased case fatality (**Figure 3**).

### Factors Associated With Case Fatality

The number of deaths (n = 44) in our study was relatively low and the number of variables included in this analysis was relatively large; therefore, the LASSO model (**Supplementary Figure 1**), in combination with clinical relevance, was used to screen the variables for logistic regression analysis. Age, sex, neutrophil count, lymphocyte count, eosinophil count, DBIL, prealbumin, creatinine, uric acid, prothrombin time, and FBG trajectory patterns were selected for univariable logistic regression analysis. Univariate logistic regression analysis showed that older age; lymphopenia; eosinophilia; an increased neutrophil count; elevated DBIL, prealbumin, creatinine, and uric acid; prolonged prothrombin time; and the high-stable FBG pattern were associated with higher case fatality (**Table 2**).

**Table 2:** Univariable and multivariable analyses for death concerning all study patients.

		Univariable analysis OR (95% CI)	*P*-value	Multivariable analysis OR (95% CI)	*P*-value
FBG trajectories	Low-stable	Ref			
	High-stable	10.02 (4.24, 23.67)	<0.0001	8.79 (2.39, 32.29)	0.0011
Age, years	≤65	Ref			
	>65	3.18 (1.61, 6.30)	0.0009		
Sex	Female	0 (ref)			
	Male	1.51 (0.75, 3.04)	0.2447		
Neutrophil count, ×10^9^ /L	≤4.7	0 (ref)			
	>4.7	19.69 (4.63, 83.71)	<0.0001	25.43 (2.07, 313.03)	0.0115
Direct bilirubin, µmol/L	≤4.6	0 (ref)			
	>4.6	12.27 (5.16, 29.18)	<0.0001	5.80 (1.72, 19.58)	0.0047
Creatinine, µmol/L	≤111	0 (ref)			
	>111	21.30 (8.15, 55.65)	<0.0001	26.69 (5.82, 122.29)	<0.0001
Prothrombin time, s	≤14.3	0 (ref)			
	>14.3	9.56 (4.52, 20.20)	<0.0001		
Lymphocyte count, ×10^9^ /L	≤0.7	16.24 (7.38, 35.75)		8.07 (2.70, 24.14)	0.0002
	>0.7	1 (ref)	<0.0001		
Uric acid, µmol/L	≤428	0 (ref)			
	>428	8.04 (2.49, 26.00)	0.0005		
Prealbumin, g/L	≤100.6	14.12 (6.22, 32.07)			
	>100.6	1 (ref)	<0.0001		
Eosinophil count, ×10^9^ /L	≤0.01	0 (ref)			
	>0.01	7.38 (2.98, 18.31)	<0.0001		

FBG, fasting blood glucose; OR, odds ratio.

Multivariable logistic regression analysis showed that increased neutrophil count (OR, 25.43; 95% CI: 2.07, 313.03; *P* = 0.0115), elevated DBIL (OR, 5.80; 95% CI: 1.72, 19.58; *P* = 0.0047), elevated creatinine (OR, 26.69; 95% CI: 5.82, 122.29; *P* < 0.0001), lymphopenia (OR, 8.07; 95% CI: 2.70, 24.14; *P* = 0.0002), and high-stable FBG pattern (OR, 8.79; 95% CI: 2.39, 32.29; *P* = 0.0011) were independent risk factors for higher case fatality in patients with COVID-19 with hyperglycemia but with no history of diabetes (**Table 2**). Finally, FBG levels were higher and tended to increase during hospitalization in non-survivors more than in survivors (*P* < 0.001, **Supplementary Figure 2**).

### The Relationship Between a Reduction in the FBG Level During Hospitalization and Prognosis

Among the 230 patients, 11 (4.78%) who had been treated with hypoglycemic therapy were all in the high-stable pattern group. There were no statistically significant differences in terms of patient’s outcomes for those treated with or without hypoglycemic therapy in the patients with a high-stable pattern (*P* = 0.7011). In 146 patients (63.48%), including 9 patients who died, final FBG levels prior to discharge or death decreased relative to their on-admission FBG, whereas 84 patients (36.52%), including 35 who died, had increased final FBG relative to their on-admission FBG. Patients with decreased FBG had a lower risk of death compared to those with increased FBG (OR, 0.092; 95% CI: 0.041, 0.205; *P <* 0.0001).

In **Supplementary Figure 3**, we present the results of associations between the glycemic variations and the evolution of biochemical parameters. There were several statistically significant correlations between the glycemic variations and routine blood tests, liver and kidney function, and coagulation indicators; however, Pearson’s correlation coefficients (*r*) were all very small (all *r* < 0.4) suggesting that the strength of these relationships was weak.

## Discussion

In this study, we analyzed clinical characteristics and outcomes in relation to 230 patients with COVID-19 and abnormal FBG but without a previous diagnosis of diabetes. In preliminary study, we found that FBG ≥7.0 mmol/L at admission could predict the risk of 28-day mortality in patients with COVID-19 without previous diagnosis of diabetes (7). It has also been reported that at-admission hyperglycemia (at-admission glycemia ≥7.78 mmol/L) is a major and independent risk factor associated with a poor prognosis in people hospitalized for COVID-19 (20). However, much of our understanding of FBG levels in patients with COVID-19 has been derived from cross-sectional data. In this study, we investigated changes in FBG in hospitalized patients throughout the course of their treatment for COVID-19. This current study extended those findings to demonstrate that not only is at-admission glycemia important, but also that certain populations, such as patients with high-stable FBG patterns, were more likely to have consistently high FBG levels, placing them at higher risk of a poor outcome than those with low FBG levels.

We observed heterogeneous FBG patterns in patients with COVID-19 during their entire hospitalization period. We identified two distinctive trajectories, namely, low-stable and high-stable patterns. The average FBG level ranged from 6.63 mmol/L to 7.54 mmol/L in patients with the low-stable pattern, whereas the average FBG level ranged from 12.59 mmol/L to 14.02 mmol/L in patients with the high-stable pattern. This finding indicated that patients with a high-stable pattern fulfilled diagnostic criteria for diabetes, which involve a patient having classic symptoms of hyperglycemia or a hyperglycemic crisis and a random plasma glucose level ≥200 mg/dl (11.1 mmol/L) (21). Our study findings showed that corticosteroid therapy was associated with patient outcomes and the FBG trajectory. It is known that corticosteroids can induce hyperglycemia, which results from impairment of multiple pathways affecting carbohydrate metabolism. Glucocorticoids cause hepatic insulin resistance, resulting in increased hepatic glucose output and induce peripheral insulin resistance, which predominantly reflects insulin action in skeletal muscle (22). The American Diabetes Association defined stress-induced hyperglycemia as >140 mg/dl (7.77 mmol/L) in patients with no previous diagnosis of diabetes (23). Although many patients have multiple episodes of abnormal FBG during hospitalization, it remains unclear whether a patient should be diagnosed with type 2 diabetes, considering the possibility of stress hyperglycemia and the effect of medications on blood glucose. In a prospective cohort study, discrete FBG trajectories were significantly associated with subsequent risk of myocardial infarction and cancer in individuals without diabetes (24, 25). In addition to diabetes, changes in FBG may also play a key role in the development of other diseases. Moreover, the FBG data were more accessible than other laboratory indicators and the relationship between FBG trajectories and disease, except diabetes, was also more closely investigated.

Individuals with the high-stable pattern were more likely to have abnormal laboratory data in relation to including inflammatory cell counts (neutrophil, lymphocyte, and monocyte counts), serum liver and kidney function indicators, and coagulation function indicators than those with the low-stable pattern (**Supplementary Table 1**). A previous study revealed that hyperglycemia (12 mmol/L), regardless of the level of insulin, activated the coagulation cascade (13). In our study, 89.29% of the patients with the high-stable pattern had high D-dimer levels, which was consistent with the results reported in the aforementioned studies. Moreover, patients with systemic inflammatory response syndrome tend to be hyperglycemic (26). One clinical trial reported that hyperglycemia also enhanced coagulation and reduced neutrophil degranulation in patients during systemic inflammation (27). These results further support our findings.

The findings of our analysis indicated that participants with a high-stable FBG pattern had a higher risk of death. These findings provide a novel insight into the long-term patterns of FBG change and highlight that, among patients with COVID-19 without diabetes, there are heterogeneous FBG trajectories. To our knowledge, no prior study has examined the potential effect of FBG trajectories on the risk of death in patients with COVID-19 and with no prior diagnosis of diabetes. Similarly, it has been reported that severe hyperglycemia after admission is a strong predictor of death among patients with COVID-19 not requiring intensive care unit admission (28), which is consistent with our results. A recent study found that patients who had higher average glucose during their first week of hospitalization were more likely to have comorbidity and abnormal laboratory markers, and were at greater risk of severe pneumonia, acute respiratory distress syndrome, and death (29). Our data further confirmed that higher FBG during the entire hospitalization was independently correlated with death. Our findings indicate that a patient’s FBG is a more sensitive marker of metabolic balance.

Results of our multivariable logistic regression analysis also showed that increased neutrophil count, elevated DBIL and creatinine, and lymphopenia were independently associated with case fatality in patients with COVID-19 and no prior diagnosis of diabetes (**Table 2**). In addition, our findings suggest that non-survivors of COVID-19 were more likely to accept IMV treatment than survivors. Moreover, a higher proportion of patients with the high-stability FBG pattern received IMV treatment than those with the low-stability FBG pattern.

Our findings provide direct evidence to support recent suggestions that regularly monitoring FBG in patients with COVID-19 is of significance in terms of public health, and for clinical diagnosis and treatment. One recent study reported that continuous glucose monitoring was more sensitive than HbA1c and fasting glucose measurements in detecting dysglycemia in a Spanish population without diabetes; therefore, continuous blood glucose monitoring could facilitate screening and prompt treatment in patients with suspected dysglycemia (27). We recommend that more attention should be paid to the trajectories of the FBG pattern throughout the disease course rather than focusing particularly on one single measurement. It has been previously reported that well-controlled blood glucose levels and maintaining glycemic variability within 3.9–10.0 mmol/L is associated with a significant reduction in composite adverse outcomes and death among patients with COVID-19 with type 2 diabetes (11). A retrospective study of 2,748 medical patients in an intensive care unit showed that blood glucose levels exceeding the upper and lower limits of the normoglycemic range (4.4–6.1 mmol/L) sustained over time, a high amplitude variation, and a high entropy of blood glucose time series were independently associated with hospital mortality (30). Therefore, these findings suggest that sustained high levels or a high amplitude variation of FBG might not be beneficial for patients with COVID-19.

Nevertheless, hypoglycemic therapy, especially insulin treatment, has remained controversial for patients with COVID-19. No significant difference was found between patients treated with hypoglycemic therapy and those who were not in the high-stable pattern group in this study, which may be due to the smaller number of patients included. However, our data showed that reducing the FBG level during hospitalization may improve prognosis, suggesting that a decreased FBG may be a protective factor. One study reported that, among 25 patients with glycemic levels >7.7 mmol/L, 10 patients treated without insulin infusion had a higher risk of severe disease, including increased mortality, compared with 15 patients treated with insulin infusion (13). However, insulin treatment has been reportedly associated with increased mortality in patients with COVID-19 and type 2 diabetes (31). However, randomized controlled studies involving tight glycemic control have reported increased incidences of hypoglycemia when targeting normoglycemia (32). Moreover, a clear association has been found between the occurrence of hypoglycemia and increased mortality in critically ill patients (32), possibly due to a lack of timely changes in medication or dose adjustment. Thus, regular FBG monitoring is vital for assisting clinicians in making treatment adjustments. For critically ill patients or those with severe sepsis, the recommended strategy for glycemic control is that insulin therapy should be started when blood glucose exceeds 180 mg/dl (10 mmol/L), with a goal of maintaining blood glucose between 144 and 180 mg/dL (8–10 mmol/L) using insulin, as necessary (33, 34). Although there are no data regarding optimal glycemic targets for inpatients with COVID-19, it is clear that extreme FBG levels can lead to poor outcomes, and that continuous blood glucose monitoring and timely and appropriate blood glucose control may contribute to positive patient outcomes.

Due to its retrospective design and the unprecedented scale of the COVID-19 pandemic, this study had several limitations. First, this was a retrospective study. Second, out study comprised a small number of participants. Because the FBG measurement time was not regular and repeat FBG examinations might have been scheduled according to the change in the patient’s condition. Third, HbA1c data, used for the assessment of glycemic status before admission, were missing; therefore, we could not distinguish whether elevated FBG levels corresponded to stress hyperglycemia or to a previous history of diabetes. Fourth, the data lacked external validation.

In conclusion, in this study, we identified two FBG trajectories and found that these patterns were significantly associated with the risk of death in patients with COVID-19 without diabetes. Monitoring FBG trajectories may provide an important approach to assist clinicians in assessing disease conditions and in adjusting medication or dosages. Future research needs to explore the key risk factors associated with elevated FBG trajectories.

## Data Availability Statement

The original contributions presented in the study are included in the article/**Supplementary Material**. Further inquiries can be directed to the corresponding author.

## Ethics Statement

Ethical approval was granted by the institutional ethics committees of Wuhan Union Hospital (No. 0036). The requirement for informed consent was waived by the Ethics Commission as described previously.

## Author Contributions

YJ designed this study. SS, SZ, YM, PM, and HL collected all the data. SS and ZW analyzed the data. SW, YM, PM, HL, and MW interpreted the data. SS and SZ drafted the initial manuscript. All authors reviewed the first draft and provided essential suggestions on revision. SS and SZ revised the final manuscript. YJ is the guarantor of this work and takes responsibility for the contents of the article. All authors contributed to the article and approved the submitted version.

## Funding

This work was supported by National Key Research and Development Project of China (No.2020YFC0844300), National Science and Technology Major Project of China (No.2019ZX09301001), and the National Natural Science Foundation of China (No.82070099; No. 82041018).

## Conflict of Interest

The authors declare that the research was conducted in the absence of any commercial or financial relationships that could be construed as a potential conflict of interest.

## Supplementary Material

Supplementary Figure 1 Variables selection using LASSO regression model. **(A)** Tuning parameters (λ) selection in LASSO model used 10-fold cross-validation *via* minimum criteria. **(B)** LASSO coefficient profile of 42 clinical features (age, sex, 8 treatments, and 32 laboratory indices).

Supplementary Figure 2 Relationship between changes in fasting blood glucose levels and the monitoring time in survivors and non-survivors.

Supplementary Figure 3 Visual correlation matrix of glycemic variations and the evolution of biochemical parameters. This figure provides a visual representation of the respective correlations as measured using Pearson’s correlation coefficients (r), based on *P *= 0.05. The color-intensity signal indicates correlation strength, whereas the blue and red colors indicate strong positive and negative correlations, respectively. * <0.05. **(A)** The metrics (columns) represent biochemical parameters in blood routine test. **(B)** The metrics (columns) represent biochemical parameters in hepatic and renal function tests. **(C)** The metrics (columns) represent biochemical parameters in coagulation function test.

## Abbreviations

FBG, fasting blood glucose; COVID-19, coronavirus disease 2019; OR, odds ratio; CI, confidence interval; GBTM, group-based trajectory modeling; DBIL, direct bilirubin.
