# Supplementary figures and images for: Association Between Longitudinal Change in Abnormal Fasting Blood Glucose Levels and Outcome of COVID-19 Patients Without Previous Diagnosis of Diabetes
Source: Front Endocrinol (Lausanne). 2021 Mar 30;12:640529. doi: 10.3389/fendo.2021.640529 (PMC8042381; doi:10.3389/fendo.2021.640529)

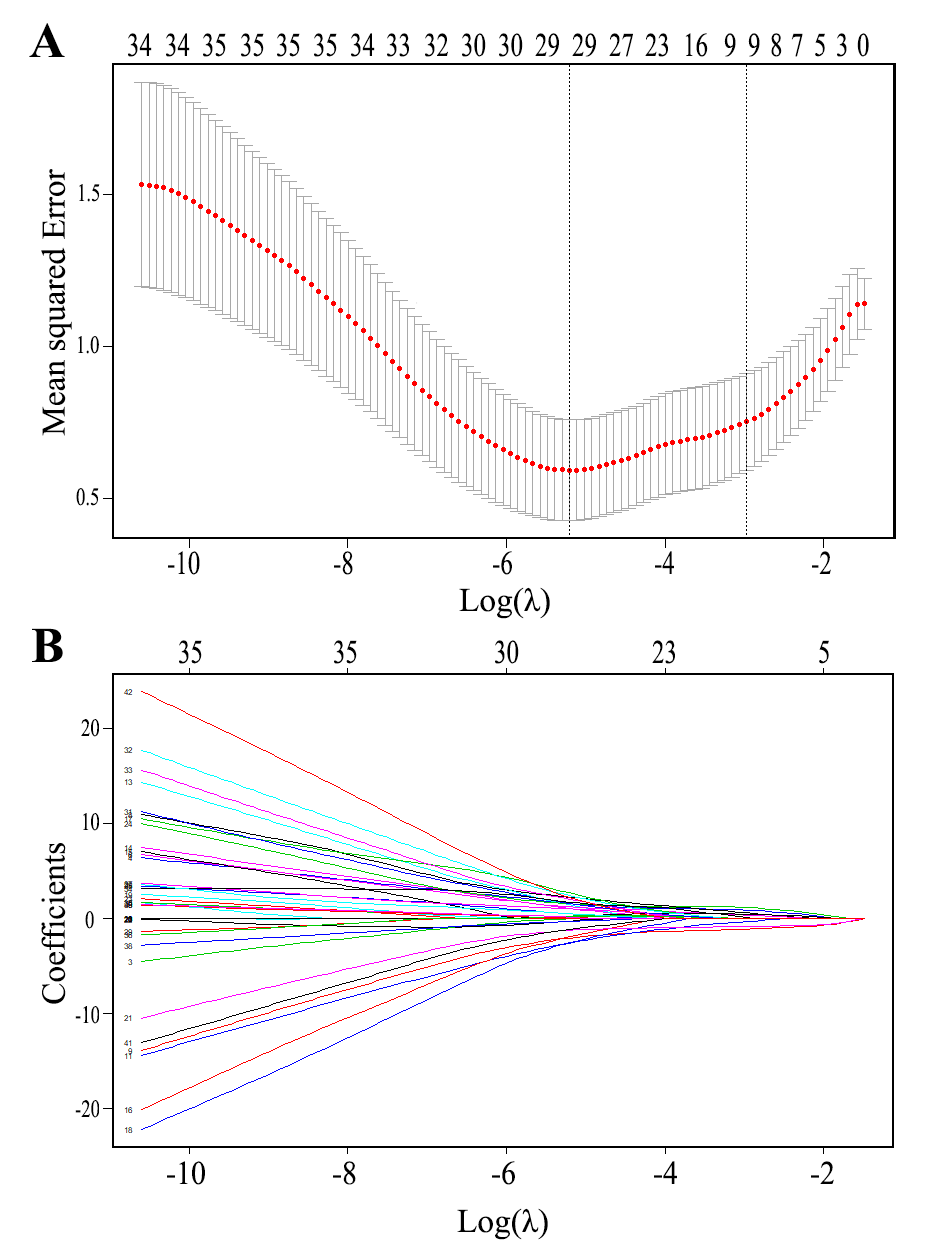

Supplement: Supplementary Figure 1 — Variables selection using LASSO regression model. (A) Tuning parameters (λ) selection in LASSO model used 10-fold cross-validation via minimum criteria. (B) LASSO coefficient profile of 42 clinical features (age, sex, 8 treatments, and 32 laboratory indices). [file Image_1.tif]

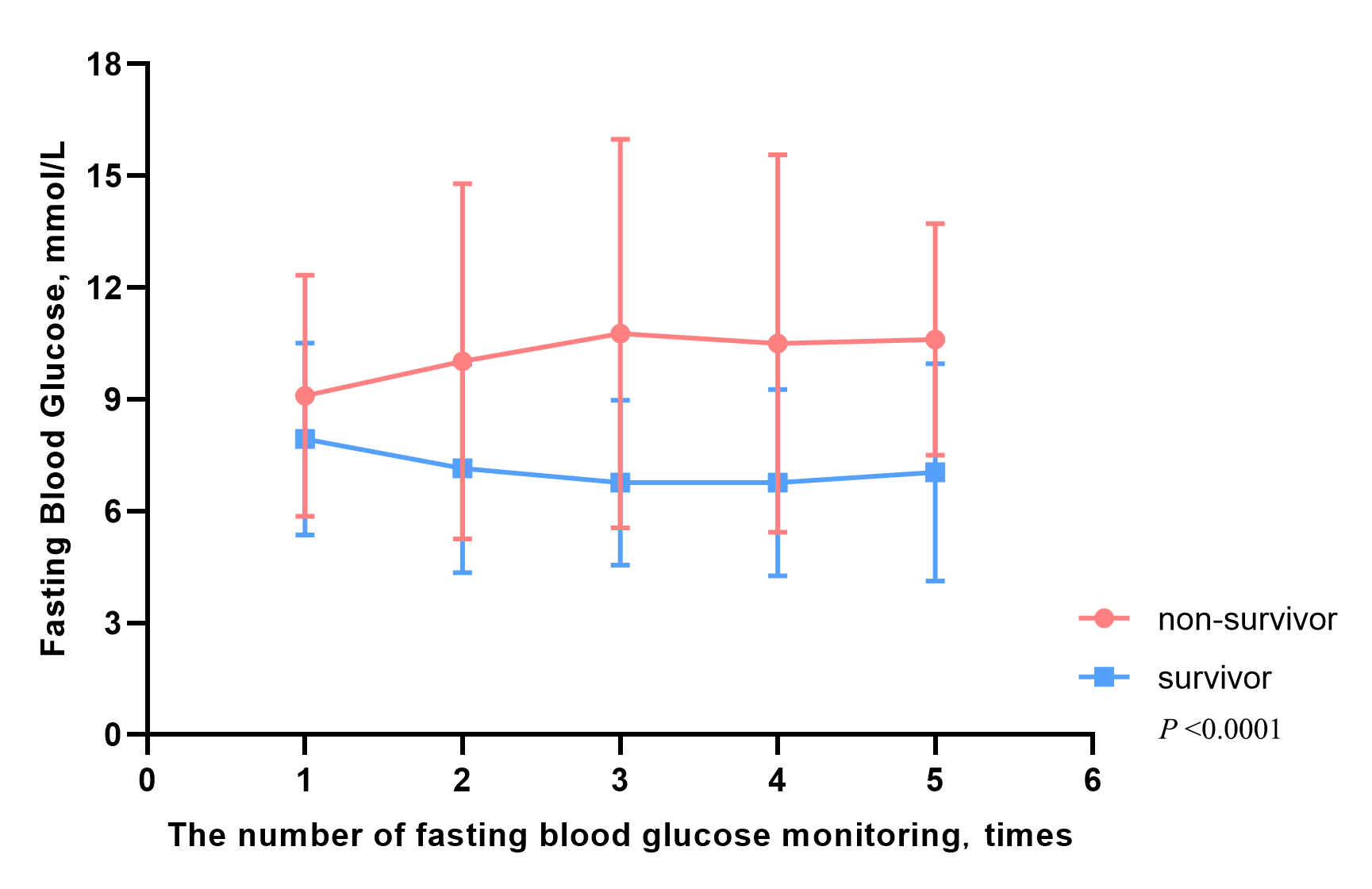

Supplement: Supplementary Figure 2 — Relationship between changes in fasting blood glucose levels and the monitoring time in survivors and non-survivors. [file Image_2.tif]

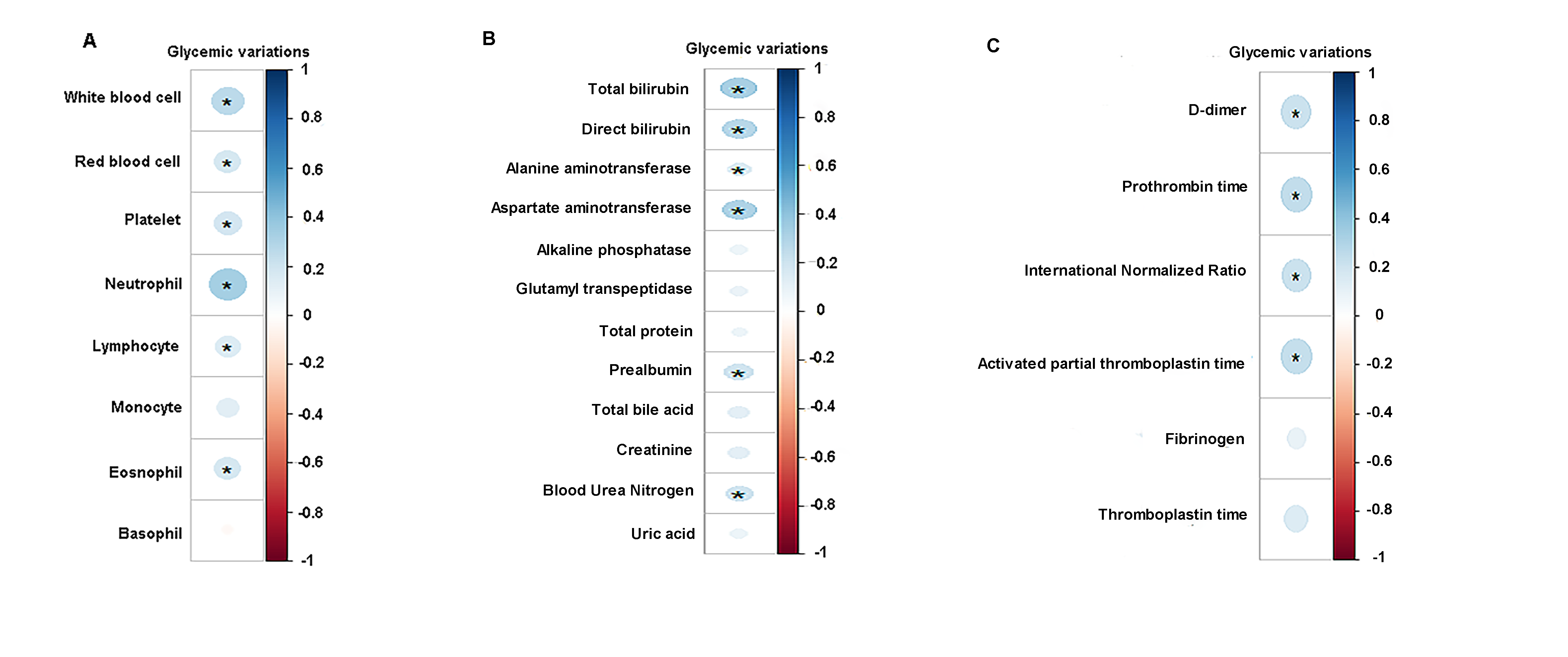

Supplement: Supplementary Figure 3 — Visual correlation matrix of glycemic variations and the evolution of biochemical parameters. This figure provides a visual representation of the respective correlations as measured using Pearson’s correlation coefficients (r), based on P = 0.05. The color-intensity signal indicates correlation strength, whereas the blue and red colors indicate strong positive and negative correlations, respectively. * <0.05. (A) The metrics (columns) represent biochemical parameters in blood routine test. (B) The metrics (columns) represent biochemical parameters in hepatic and renal function tests. (C) The metrics (columns) represent biochemical parameters in coagulation function test. [file Image_3.tif]
