# Supplementary material for: Association Between Longitudinal Change in Abnormal Fasting Blood Glucose Levels and Outcome of COVID-19 Patients Without Previous Diagnosis of Diabetes
Source: Front Endocrinol (Lausanne). 2021 Mar 30;12:640529. doi: 10.3389/fendo.2021.640529 (PMC8042381; doi:10.3389/fendo.2021.640529)
Supplement: Supplementary file 4 [file Table_1.docx]

Supplementary Material

## Supplementary Table

**Supplementary Table 1 Baseline clinical characteristics and laboratory finding concerning all study patients during hospitalization**

|  | Low-stable  (n=202) | High-stable  (n=28) | *P*-value |
| --- | --- | --- | --- |
| Age, years |  |  |  |
| Median, (*IQR*) | 63.00 (53.00～69.00) | 69.50 (57.50～71.50) | 0.0359 |
| ≤65, n (%) | 127/202 (62.87) | 9/28 (32.14) | 0.0019 |
| >65, n (%) | 75/202 (37.13) | 19/28 (67.86) |  |
| Sex |  |  |  |
| Female, n (%) | 81/202 (40.10) | 10/28 (35.71) | 0.6566 |
| Male, n (%) | 121/202 (59.90) | 18/28 (64.29) |  |
| Onset symptoms |  |  |  |
| Fever, n (%) | 162/194 (83.51) | 21/24 (87.50) | 0.7738 |
| Pharyngalgia, n (%) | 7/179 (3.91) | 1/22 (4.55) | >0.9999 |
| Fatigue, n (%) | 88/184 (47.83) | 12/22 (54.55) | 0.5512 |
| Muscular soreness, n (%) | 36/181 (19.89) | 6/22 (27.27) | 0.4195 |
| Cough, n (%) | 140/187 (74.87) | 18/23 (78.26) | 0.7219 |
| Expectoration, n (%) | 70/182 (38.46) | 14/23 (60.87) | 0.0395 |
| Hemoptysis, n (%) | 6/180 (3.33) | 0/22 (0.00) | 0.5965 |
| Dyspnea, n (%) | 72/181 (39.78) | 9/23 (39.13) | 0.9523 |
| Rhinorrhea, n (%) | 3/179 (1.68) | 0 | >0.9999 |
| Nausea, n (%) | 15/181 (8.29) | 2/22 (9.09) | >0.9999 |
| Vomiting, n (%) | 12/181 (6.63) | 2/22 (9.09) | 0.6525 |
| Stomachache, n (%) | 3/179 (1.68) | 1/24 (4.17) | 0.577 |
| Diarrhea, n (%) | 26/181 (14.36) | 3/23 (13.04) | >0.9999 |
| Poor appetite, n (%) | 32/181 (17.68) | 4/22 (18.18) | 0.9536 |
| Headache, n (%) | 11/179 (6.15) | 0 | 0.6181 |
| Delirium, n (%) | 0 | 0 | / |
| Chest distress, n (%) | 57/181 (31.49) | 9/23 (39.13) | 0.4608 |
| Non-symptoms, n (%) | 0 | 0 | / |
| Comorbidity |  |  |  |
| Chronic obstructive pulmonary disease, n (%) | 3/175 (1.71) | 0 | >0.9999 |
| Asthma, n (%) | 3/175 (1.71) | 0 | >0.9999 |
| Hypertension, n (%) | 68/179 (37.99) | 6/23 (26.09) | 0.2648 |
| Chronic cardiac disease, n (%) | 25/178 (14.04) | 2/23 (8.70) | 0.7458 |
| Anemia, n (%) | 1/174 (0.57) | 0 | >0.9999 |
| Chronic kidney disease, n (%) | 9/174 (5.17) | 0 | 0.6021 |
| Chronic hepatic disease, n (%) | 5/174 (2.87) | 0 | >0.9999 |
| Cerebrovascular disease, n (%) | 5/174 (2.87) | 1/23 (4.35) | 0.5301 |
| Malignant disease, n (%) | 9/174 (5.17) | 4/23 (17.39) | 0.0265 |
| Respiratory support |  |  |  |
| Invasive mechanical ventilation, n (%) | 18/202 (8.91) | 8/28 (28.57) | 0.0021 |
| Treatment |  |  |  |
| Traditional Chinese medicine, n (%) | 115/202(56.93) | 17/28(60.71) | 0.7043 |
| Antiviral therapy, n (%) | 183/202(90.59) | 27/28(96.43) | 0.5035 |
| Antibiotic therapy, n (%) | 151/202(74.75) | 24/28(85.71) | 0.2993 |
| Corticosteroid, n (%) | 75/202(37.13) | 18/28(64.29) | 0.0061 |
| Intravenous immunoglobin, n (%) | 53/202(26.24) | 5/28(17.86) | 0.3386 |
| Antihypertensive medicine, n (%) | 42/202(20.79） | 4/28（14.29） | 0.5792 |
| Lipid-lowering therapy, n (%) | 10/202（4.95） | 1/28（3.57） | >0.9999 |
| Hypoglycemic therapy n (%) | 0 | 11/28（39.29） | <0.0001 |
| Laboratory findings |  |  |  |
| White blood cell count, ×10^9^ /L |  |  | 0.0026 |
| ≤5.6, n (%) | 62/202 (30.69) | 1/28 (3.57) |  |
| >5.6, n (%) | 140/202 (69.31) | 27/28 (96.43) |  |
| Red blood cell count, ×10^12^ /L |  |  | 0.3509 |
| ≤4.0, n (%) | 111/202 (54.95) | 18/28 (64.29) |  |
| >4.0, n (%) | 91/202 (45.05) | 10/28 (35.71) |  |
| Platelet count, ×10^9^ /L |  |  | <0.0001 |
| ≤136, n (%) | 30/202 (14.85) | 15/28 (53.57) |  |
| >136, n (%) | 172/202 (85.15) | 13/28 (46.43) |  |
| Neutrophil count, ×10^9^ /L |  |  | <0.0001 |
| ≤4.7, n (%) | 91/202 (45.05) | 1/28 (3.57) |  |
| >4.7, n (%) | 111/202 (54.95) | 27/28 (96.43) |  |
| Lymphocyte count, ×10^9^ /L |  |  | 0.0001 |
| ≤0.7, n (%) | 46/202 (22.77) | 16/28 (57.14) |  |
| >0.7, n (%) | 156/202 (77.23) | 12/28 (42.86) |  |
| Monocyte count, ×10^9^ /L |  |  | 0.0003 |
| ≤0.2, n (%) | 8/202 (3.96) | 6/28 (21.43) |  |
| >0.2, n (%) | 194/202 (96.04) | 22/28 (78.57) |  |
| Eosinophil count, ×10^9^ /L |  |  | 0.4199 |
| ≤0.01, n (%) | 19/202 (9.41) | 4/28 (14.29) |  |
| >0.01, n (%) | 183/202 (90.59) | 24/28 (85.71) |  |
| Basophil count, ×10^9^ /L |  |  | 0.7339 |
| ≤0.01, n (%) | 34/202 (16.83) | 4/28 (14.29) |  |
| >0.01, n (%) | 168/202 (83.17) | 24/28 (85.71) |  |
| Total bilirubin, µmol/L |  |  | 0.0202 |
| ≤12.3, n (%) | 105/202 (51.98) | 8/28 (28.57) |  |
| >12.3, n (%) | 97/202 (48.02) | 20/28 (71.43) |  |
| Direct bilirubin, µmol/L |  |  | 0.0061 |
| ≤4.6, n (%) | 127/202 (62.87) | 10/28 (35.71) |  |
| >4.6, n (%) | 75/202 (37.13) | 18/28 (64.29) |  |
| Alanine aminotransferase, U/ |  |  | 0.3277 |
| ≤23.3, n (%) | 24/202 (11.88) | 1/28 (3.57) |  |
| >23.3, n (%) | 178/202 (88.12) | 27/28 (96.43) |  |
| Aspartate aminotransferase, U/L |  |  | 0.2678 |
| ≤27.2, n (%) | 56/202 (27.72) | 5/28 (17.86) |  |
| >27.2, n (%) | 146/202 (72.28) | 23/28 (82.14) |  |
| Alkaline phosphatase, U/L |  |  | 0.0288 |
| ≤53, n (%) | 43/201 (21.39) | 1/27 (3.70) |  |
| >53, n (%) | 158/201 (78.61) | 26/27 (96.30) |  |
| Glutamyl transpeptidase, U/L |  |  | 0.1119 |
| ≤27.3, n (%) | 50/201 (24.88) | 3/27 (11.11) |  |
| >27.3, n (%) | 151/201 (75.12) | 24/27 (88.89) |  |
| Total protein, g/L |  |  | <0.0001 |
| ≤58.6, n (%) | 42/202 (20.79) | 17/28 (60.71) |  |
| >58.6, n (%) | 160/202 (79.21) | 11/28 (39.29) |  |
| Globulin, g/L |  |  | 0.8587 |
| ≤30.5, n (%) | 83/202 (41.09) | 12/28 (42.86) |  |
| >30.5, n (%) | 119/202 (58.91) | 16/28 (57.14) |  |
| Prealbumin, mg/L |  |  | <0.0001 |
| ≤100.6, n (%) | 24/184 (13.04) | 13/26 (50.00) |  |
| >100.6, n (%) | 160/184 (86.96) | 13/26 (50.00) |  |
| Albumin, g/L |  |  | 0.0015 |
| ≤33.8, n (%) | 136/202 (67.33) | 27/28 (96.43) |  |
| >33.8, n (%) | 66/202 (32.67) | 1/28 (3.57) |  |
| Total bile acid, µmol/L |  |  | 0.9172 |
| ≤3.5, n (%) | 84/201 (41.79) | 11/27 (40.74) |  |
| >3.5, n (%) | 117/201 (58.21) | 16/27 (59.26) |  |
| Creatinine, µmol/L |  |  | 0.9172 |
| ≤111, n (%) | 84/201 (41.79) | 11/27 (40.74) |  |
| >111, n (%) | 117/201 (58.21) | 16/27 (59.26) |  |
| Blood Urea Nitrogen, mmol/L |  |  | 0.0037 |
| ≤8.2, n (%) | 159/202 (78.71) | 15/28 (53.57) |  |
| >8.2, n (%) | 43/202 (21.29) | 13/28 (46.43) |  |
| Uric acid, µmol/L |  |  | 0.2002 |
| ≤428, n (%) | 192/202 (95.05) | 25/28 (89.29) |  |
| >428, n (%) | 10/202 (4.95) | 3/28 (10.71) |  |
| Creatine kinase, U/L |  |  | 0.0846 |
| ≤83, n (%) | 99/188 (52.66) | 9/26 (34.62) |  |
| >83, n (%) | 89/188 (47.34) | 17/26 (65.38) |  |
| D-dimer, µg/mL |  |  | 0.0016 |
| ≤0.97, n (%) | 81/195 (41.54) | 3/28 (10.71) |  |
| >0.97, n (%) | 114/195 (58.46) | 25/28 (89.29) |  |
| Prothrombin time, s |  |  | 0.0005 |
| ≤14.3, n (%) | 146/195 (74.87) | 12/28 (42.86) |  |
| >14.3, n (%) | 49/195 (25.13) | 16/28 (57.14) |  |
| International Normalized Ratio |  |  | 0.0168 |
| ≤1.1, n (%) | 129/195 (66.15) | 12/28 (42.86) |  |
| >1.1, n (%) | 66/195 (33.85) | 16/28 (57.14) |  |
| Activated partial thromboplastin time, s |  |  | 0.0042 |
| ≤40.2, n (%) | 150/198 (75.76) | 14/28 (50.00) |  |
| >40.2, n (%) | 48/198 (24.24) | 14/28 (50.00) |  |
| Thromboplastin time, s |  |  | 0.0176 |
| ≤16.5, n (%) | 148/190 (77.89) | 16/28 (57.14) |  |
| >16.5, n (%) | 42/190 (22.11) | 12/28 (42.86) |  |
| Fibrinogen, g/L |  |  | 0.5803 |
| ≤4.1, n (%) | 110/198 (55.56) | 14/28 (50.00) |  |
| >4.1, n (%) | 88/198 (44.44) | 14/28 (50.00) |  |
| C-reactive protein, mg/L |  |  | 0.0007 |
| ≤21.4, n (%) | 81/197 (41.12) | 2/27 (7.41) |  |
| >21.4, n (%) | 116/197 (58.88) | 25/27 (92.59) |  |
| Erythrocyte sedimentation rate, mm/h |  |  | 0.6157 |
| ≤22, n (%) | 8/79 (10.13) | 2/12 (16.67) |  |
| >22, n (%) | 71/79 (89.87) | 10/12 (83.33) |  |

Data are median (*IQR*) or n (%). *P*-values were calculated by using χ^2^ test, Cochran–Mantel–Haenszel χ^2^ test, Fisher’s exact test or Wilcoxon rank-sum test, as appropriate.

*IQR*: interquartile range
